# Microfluidic Gastrointestinal Cell Culture Technologies—Improvements in the Past Decade

**DOI:** 10.3390/bios14090449

**Published:** 2024-09-19

**Authors:** Adrian J. T. Teo, Siu-Kin Ng, Kaydeson Khoo, Sunny Hei Wong, King Ho Holden Li

**Affiliations:** 1 School of Mechanical and Aerospace Engineering, Nanyang Technological University, Singapore 639798, Singapore; adrian.teojt@ntu.edu.sg (A.J.T.T.); kayd0002@e.ntu.edu.sg (K.K.); 2 Lee Kong Chian School of Medicine, Nanyang Technological University, Singapore 308232, Singapore; siukin.ng@ntu.edu.sg (S.-K.N.); sunny.wong@ntu.edu.sg (S.H.W.); 3 Department of Gastroenterology and Hepatology, Tan Tock Seng Hospital, Singapore 308433, Singapore

**Keywords:** microfluidics, cell culture, gastrointestinal tract, gut-on-a-chip

## Abstract

Gastrointestinal cell culture technology has evolved in the past decade with the integration of microfluidic technologies, bringing advantages with greater selectivity and cost effectiveness. Herein, these technologies are sorted into three categories, namely the cell-culture insert devices, conventional microfluidic devices, and 3D-printed microfluidic devices. Each category is discussed in brief with improvements also discussed here. Introduction of different companies and applications derived from each are also provided to encourage uptake. Subsequently, future perspectives of integrating microfluidics with trending topics like stool-derived in vitro communities and gut–immune–tumor axis investigations are discussed. Insights on modular microfluidics and its implications on gastrointestinal cell cultures are also discussed here. Future perspectives on point-of-care (POC) applications in relations to gastrointestinal microfluidic devices are also discussed here. In conclusion, this review presents an introduction of each microfluidic platform with an insight into the greater contribution of microfluidics in gastrointestinal cell cultures.

## 1. Introduction

Microfluidics is a field of study that focuses on understanding and improving the operation and manipulation of fluids at a microscale level. The establishment of said theories accordingly lead to the development of miniaturized technologies that can directly trigger certain responses within small devices [1]. This allows minute volumes of fluids of up to femtoliters to be handled effectively within prefabricated microchannels. Such benefits entail its strong uptake in various industries [2], especially in the biomedical industry, where sample volumes and reagents have to be kept to a minimum for cost optimization purposes. Apart from this, the precise control and manipulation over minute volumes of fluid has also enabled its use for applications such as drug testing where accurate dosing is pivotal [3]. Transport of such devices for on-site evaluations is also possible due to their small size [4], while also being highly versatile and adaptable for wide usage [5]. Through additional optimization and automation steps, manual intervention of users may also be relegated while still ensuring efficiency and consistency in experimental conditions [6].

Microfluidic technology finds application in the biomedical field in the areas of drug discovery, microbiome analysis, and cell analysis [7,8]. In comparison to the conventional animal testing models, it has less administrative challenges with no animal rights’ clearances needed [9], is less costly with less laboratory infrastructural requirements, and does not need highly skilled staffing [10]. A typical animal model requires proper animal facilities for rearing the animals, which may range from small insects to larger animals like rats, rabbits and even primates. Staff would also need to be organized to ensure proper caretaking of the animals throughout the duration of breeding. Finally, drugs or culling methods would also need to be administered in accordance with animal cruelty rights of the designated country. The use of microfluidic technology to replace animal testing models would thus also speed up research while enabling a more efficient use of manpower and space in this aspect.

Here, microfluidics is often used as a platform for cell cultures as bioreactors, lab-on-a-chip, and organ-on-a-chip devices. Prior to this, bioreactors comprising of roller bottles, packing beds, and stirrer tanks were used [11]. These platforms enabled large-scale production of culture-based vaccines at industrial scales and increased process efficiency with continuous operation. Single-use bioreactors were also developed out of disposable plastics as compared with stainless-steel tanks for smaller-scale productions, of volumes up to 2000 L depending on the type [11]. A recent publication also discussed the use of such bioreactors in organoid culture [12]. Specific advantages and limitations on the use of microfluidics as a bioreactor platform were also briefly discussed. However, due to the scope of that review, it was observed that the points may be limited to organoid culture which may not represent the full spectrum of cell culturing.

In principle, almost any type of human cells can be cultured on a chip. These cells can be classified as primary cells, transformed cells, or self-renewing cells [13]. Primary cells are obtained directly from human samples or biopsies, transformed cells being generated either naturally or genetically manipulated with immortalized cell lines, and self-renewing cells are stem cells that can differentiate into other cell types. These cells are accordingly derived from different parts of the body like the brain (neuronal cells, dendritic cells) [14], liver (hepatocytes HepG2, liver nonparenchymal cells) [15,16], lung (lung cancer cell line SPCA-1, fibroblasts HFL1) [17], and intestine (intestinal epithelial cells Caco-2, vascular endothelial cells) [18]. Understanding that these parts are all working in tandem and interacting with each other, multiple reviews have also been carried out to discuss the different interactions and outcomes. Arumugasaamy et al. [19] discussed the transport across the body’s natural epithelial barrier along the skin, lungs, intestines, and placenta while acknowledging interactions with the blood–brain barrier for some of these parts. Guo et al. [20] described the interaction of the gastrointestinal system with the brain, liver, kidneys, and lungs in detail. However, it was mainly limited to organ-on-a-chip microfluidic models, which only make up a small group of “microfluidic systems”. Another review by Kimura et al. [21] discussed microfluidic multi-organ systems for drug discovery based on works conducted prior to 2017. As such, the need for an updated review of the improvements in microfluidic cell culture technologies arises.

Through comprehensive research across the different human systems, it was observed that the human gastrointestinal (GI) tract plays an important role in the human body due to its extensive surface area and the complexity of its host cells. The internal surface area of the GI tract, enhanced by structures like villi and microvilli, is estimated to be around 30 to 40 square meters, allowing for efficient nutrient absorption and interaction with a vast array of substances. The GI tract serves as a vital epithelial barrier, facilitating the selective transport of nutrients and water into the bloodstream and preventing infections by harmful pathogens. Moreover, the GI tract is the body’s largest immune organ [22] and supports the largest ecosystem in the body. This diverse microbial community includes trillions of bacteria, viruses, fungi, and other microorganisms that play essential roles in digestion, metabolism, and immune function. The intricate host–microbe interactions within the GI tract are essential for maintaining various aspects of health. They influence not only digestive health but also immune function, metabolic processes, and even neurological health [23]. Understanding and nurturing these interactions [24] can lead to improved health outcomes and the prevention of many diseases. There is evidence that certain commensals or probiotics could enhance intestinal barrier function in vivo while also delaying the injury derived from EIEC infection by almost 18 h from infection [25,26]. Min et al. also used *Lactobacillus rhamnosus* GG and VSL#3 in a microfluidic device to observe an increase in barrier integrity, improvement in barrier function, and a decrease in inflammation [27]. Further studies related to drug testing and microbial community interactions in response to drugs, toxins, and intestinal diseases have also been demonstrated accordingly [28,29]. The consumption of probiotics like HY7715 [30], HY8002 [30], VSL#3 [31], Synbiotic SYN [31], *Lactobacillus rhamnosus* GG [32], and bifidobacteria [32] have been shown to help maintain a healthy intestine. The effects of probiotics against pathogenic bacteria invasions, immune functions, and protection mechanism have also been investigated in vitro [26,33]. As observed there, microfluidic devices have also proved useful in studying the interactions between the host cells and a probiotics co-culture within the GI tract [25,30,32].

**Figure 1:**
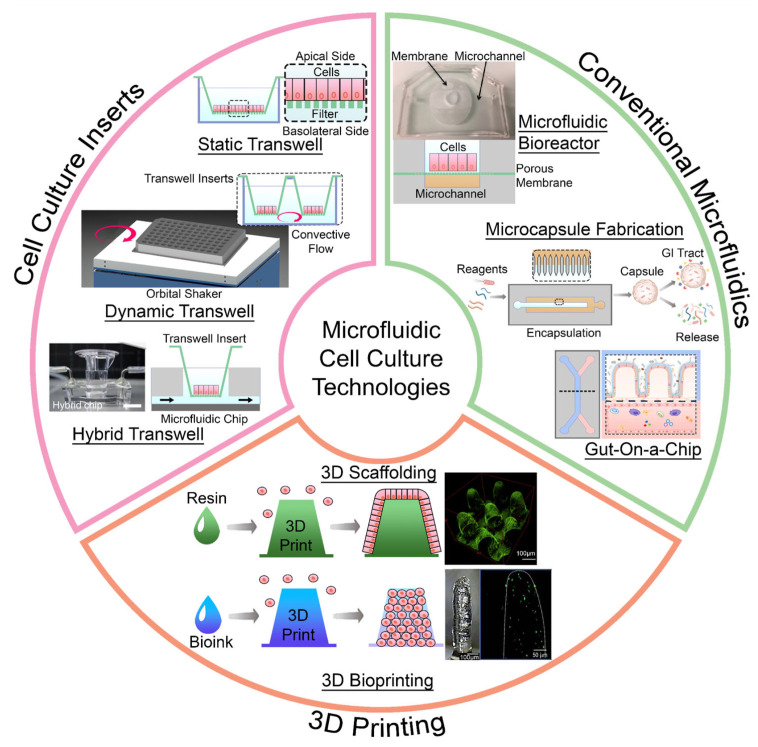
Summary of cell culture technologies developed in the past decade for gastrointestinal applications categorized according to cell culture inserts [13,37,38], conventional microfluidics [39,40,41] and 3D printing methods where the villus-like structures were created either by creating a scaffold where cells are seeded on it or bioprinting the entire structure with cells integrated within [42,43]. Reproduced with permission from [13], 2022, MDPI AG. Reproduced with permission from [39], 2020, MDPI AG. Adapted with permission from [40], 2024, American Chemical Society. Reproduced with permission from [41], 2023, Elsevier. Reproduced with permission from [42], 2019, Elsevier. Reproduced with permission from [43], 2018, Elsevier.

The uptake of microfluidics in biomedical applications have not only brought forth great research results but also resulted in the establishment of different companies selling commercial products like Illumina [34], Mimetas [35] and Emulate [36]. Apart from commercial products, a number of different microfluidic platforms have also been developed for similar applications. Here, we categorize them according to the types of devices in accordance with the type of platforms, i.e., cell culture insert types, conventional microfluidic types, and 3D-printed types (see Figure 1). In this review, we thus aim to provide insights on each type of platform while providing a brief review of the works previously published by others. The cell culture insert section further explores the models regarding 2D and 3D cell culture with static, dynamic, and hybrid transwell settings. The conventional microfluidics section explores its primary categories, including bioreactors, gut-on-a-chip (GOC) devices, which consist of 2D and 3D cell cultures, and lastly, microfluidic capsules. The 3D-printing section looks at the application of scaffold fabrication and 3D bioprinting. The integration of microfluidic technologies on trending topics like stool-derived in vivo communities and gut–immune–tumor issues are also discussed alongside modular microfluidics integration and point-of-care (POC) applications.

## 2. Microfluidic Technologies

### 2.1. Cell Culture Inserts

One common platform observed in the above-mentioned applications involves the use of cell culture inserts. These types of devices are also commonly referred as static transwell models/systems in multiple reviews [38,44,45,46]. A static transwell system typically consists of a transwell insert permeable membrane separating two distinct compartments, the upper and lower compartments. The upper compartment, otherwise known as apical chamber, represents the luminal side or external environment. The lower compartment, or the basolateral chamber, represents the side facing the bloodstream or tissue in vitro [47]. Cells are cultured on the apical chamber side of the membrane with the membrane acting as a support and barrier that prevents cell migration [48,49]. The membranes are generally made of synthetic biocompatible materials such as polycarbonate (PC), polyester (PET), and polytetrafluoroethylene (PTFE) [50], with occasional 3D-printed materials like gelatin [51]. The porosity of the membrane also permits the exchange of nutrients, gasses, and molecules between both compartments [44]. This platform thus provides a convenient in vivo platform to test physiological conditions and investigate complex biological processes. A list of companies producing such cell culture inserts includes ThermoFisher [52], Corning [53], and cellQART [54]. These inserts can be used alongside their well-plate counterparts or separately integrated into customized devices. This method is commonly found in biological laboratories due to its easy assembly and usage of off-the-shelf products by the companies mentioned above.

**Figure 2:**
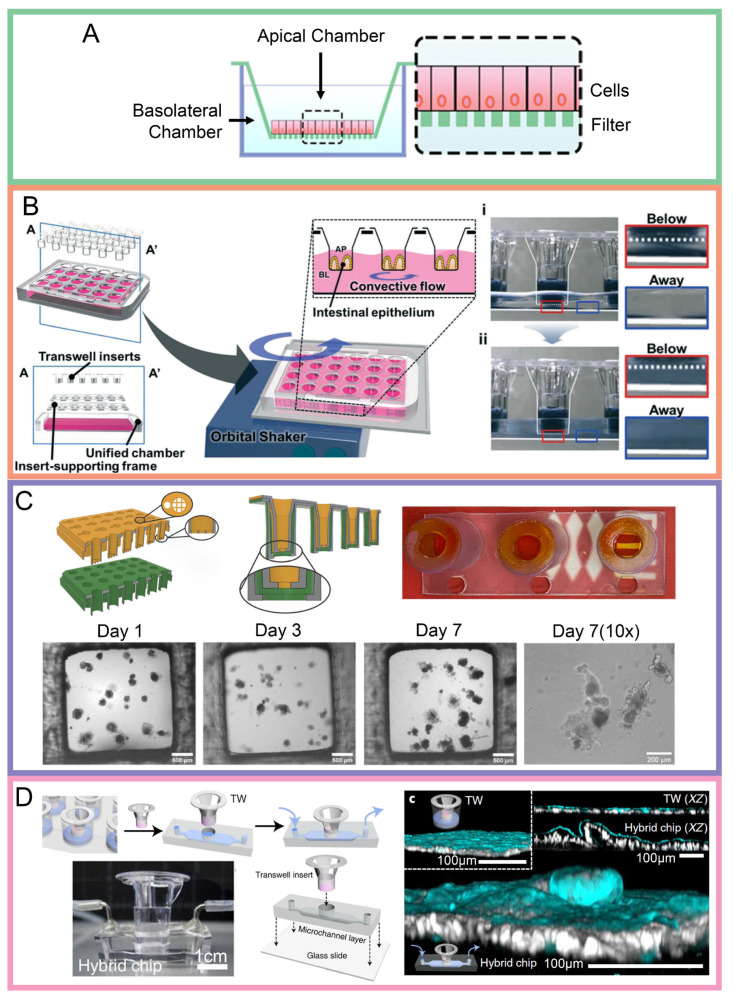
(**A**) Schematic diagram of typical static transwell setup [63]. (**B**) Schematic diagram of dynamic transwell setup with convective flow due to physical shaking of well plate [38]. Inserts show the effects of (**i**) stationary and (**ii**) induced flow with better mixes for the latter as judged by the red and blue boxes. Reproduced with permission from [38], 2021, Royal Society of Chemistry. (**C**) 3D printed transwell device showing successful growth of Caco-2 cells using a gelatin based membrane after seven days of culturing [51] Scale bars for Day 1–7 represent 500 µm, 200 µm for Day 7 (10×). Reproduced with permission from [51], 2022, Open Access. (**D**) Hybrid transwell microfluidic device showing single layer of organoid-derived epithelial cells within the transwell [13]. Adapted with permission from [13], 2022, Springer Nature.

***Static transwell model***: This model (see Figure 2A) has the advantage of being simple, reproducible, and scalable, which makes it a practical option for researchers from various fields [38]. One of the most outstanding characteristics of the static transwell model is the opportunity to analyze the different features of cell behavior, such as barrier function, transport mechanisms, and cell–cell interactions, under stable conditions [19]. A full list of advantages and disadvantages are given in Table 1. Human gut organoid-derived cultures have been adapted to 2D monolayer cultures and have been utilized to replicate diverse biological processes, encompassing infectious disease and abnormalities in barrier function [55,56]. Schweinlin et al. cultured a small intestinal organoid that utilized a decellularized small intestinal submucosa as a scaffold with results revealing that both the CYP3A4 and P-glycoprotein levels were far higher than those grown within a conventional static bioreactor [57]. Integrating previous knowledge of growing organoids on hydrogels on plates [58,59], intestinal organoids were also successfully cultured in 2019 with improvements, by loading inserts with Matrigel to enable 3D cell cultures instead of the conventional 2D monolayer cell cultures [60]. This platform effectively enabled the study of barrier function, permeability, and drug responses for applications like drug development, disease modeling, and personalized medicine [16,45,61], making it a physiologically relevant model. Culturing Caco-2 cells in a static transwell model has thus become the standard regulatory model used for drug bioavailability assays [61] with no additional ethical approval required as the cells are commercially available [62].

Three-dimensional cell cultures, being more physiologically relevant [49,63,64,65], have also made use of these cell culture inserts to interconnect tissues by co-culturing multiple cell lines and creating 3D tissues such as organoid cultures with hydrogels [60,66,67]. One such example would be the use of a Repligut cell culture platform by Pike et al. [68] to derive the GI epithelium using human primary stem cells. Custom-designed 3D-printed insert plates with multi-layer insert plates were previously designed to interconnect various tissues from the intestinal layer, vascular layer, and hepatic layer by Dufva et al. [51,69] (see Figure 2C). It is also believed that 3D cultures would provide deeper insights as compared with 2D cultures into processes such as drug permeability, drug toxicity screening, infectious disease pathogenesis due to this multi-layered ability.

Despite its multiple advantages and versatility, the static transwell system unfortunately has its own set of disadvantages. As it is a static system, it lacks the ability to produce mechanical cues such as fluid flow or shear stress, which plays a crucial role (See Table 1). To address these limitations, improved versions have been developed in the form of dynamic and hybrid transwell systems with mechanical cues.

***Dynamic transwell model***: A dynamic transwell model (see Figure 2B) is an advanced system that integrates traditional transwell models with fluidic flow, creating a more physiologically relevant environment for studies and experiments. In a typical experimental setup, additional equipment like an orbital shaker [38] (see Figure 2B) or additional pumps [14] are used to induce flow, making the microenvironment within the wells dynamic [70]. The fluidic flow creates shear stress on the cells, which is a crucial factor to be considered as it influences cellular behavior and cell differentiation. The dynamic microenvironment thus also enables a more physiologically relevant study for nutrient adsorption, cell function, drug transportation, and infectious diseases in real time, mentioned in Table 1.

A BASIN platform developed by Chong et al. [38] was essentially a dynamic transwell system that enhanced the physiological relevance of Caco-2 epithelial models by introducing a unified chamber with orbital shaking. This setup generated a basolateral convective flow while promoting the dispersion of Wnt inhibitors and supporting the morphogenesis of a 3D villilike structure. Another study conducted by Santbergen et al. [71] used dynamic flow-through transwell systems where Caco-2 and HT29-MTX cells were co-cultured and connected to the UPLC-QTOF-MS through switchable valves to study the drug permeability and biotransformation. This system combined real-time monitoring with a high-throughput analysis and provided continuous, automated data collection. This dynamic setup enhanced physiological relevance, mimicked the intestinal flow, and allowed for the simultaneous measurement of drug transport and metabolite formation, as mentioned in Table 1.

***Hybrid transwell models***: Building on the benefits of both static and dynamic transwell systems, a hybrid transwell system has been introduced (see Figure 2D). This is a system which incorporates transwell inserts with microfluidic technology. A hybrid transwell model by Shin et al. [13] successfully enhanced the 3D epithelial morphogenesis by removing morphogen antagonists from the basolateral compartment. It also facilitated scalable cell culture in multi-well porous inserts in a 24-, 96- or 384-well plate with controlled fluid dynamics. This hybrid chip was more scalable, accessible, and provided a less complex culture environment compared to a gut-on-a-chip system. Although there were multiple advantages, there were disadvantages too. In the setup, the apical compartment remained static, which failed to simulate dynamic conditions, which may reduce physiological relevance. Additionally, the polyester membrane of the transwell insert was not elastic and therefore could not produce the intestinal peristalsis motion. Therefore, future improvements of hybrid systems can incorporate dynamic apical compartment or elastic membrane to better mimic the intestine in vivo.

When a comparison is made between a pure dynamic and a hybrid transwell system, the latter combines the dynamic feature of a continuous fluidic flow with the simplicity and scalability of a traditional static transwell. Hence, each system has its own advantages and disadvantages and should be chosen based on the experimental needs and goals. Apart from transwell systems, greater interest has arisen in some aspects of microfluidic devices like microfluidic bioreactors, microcapsule production devices, and gut-on-a-chip devices, which are covered in the next section.

**Table 1:** Advantages and disadvantages of cell culture insert types [13,38,71,72,73,74].

Advantages	Disadvantages
Static Transwell Models Simple setup High scalability High reproducibility Easy recovery of cells Physiologically relevant Dynamic Transwell Models Real-time monitoring Improved cell functionality Improved physiological mimicry Hybrid Transwell Models Real-time monitoring Improved cell functionality Improved physiological mimicry	Static Transwell Models Mainly static Limited physiological correlation Cultures may have low repeatability Material may affect structure formation Inability to provide mechanical influences Dynamic Transwell Models Increased cost Increased complexity Require technical expertise Hybrid Transwell Models Increased cost Increased complexity Require technical expertise

### 2.2. Conventional Microfluidic Devices

The microfluidic devices mentioned in this section typically consist of microchannels which are manufactured on substrates like silicon and glass and polymers such as polymethyl methacrylate (PMMA) or polydimethylsiloxane (PDMS) [75]. These microchannels are intricately patterned using techniques such as photolithography, soft lithography, or micro-machining to produce multiple features within a single chip, resulting in a highly versatile platform for biomedical experiments [58,76]. Although such fabrication methods (See Supplementary Materials Table S1) may seem daunting to the inexperienced, the benefits of using such devices far outweigh the costs [77]. Trapping and culturing of cells with peristaltic motions and co-culturing with different microbiomes can be carried out using pressure-driven or flow-driven mechanisms [78]. This enables its use as a platform for the development of bioreactors and organ-on-a-chip devices. We accordingly categorized these microfluidic devices into three types: microfluidic bioreactors, microcapsule production devices, and gut-on-a-chip devices.

**Figure 3:**
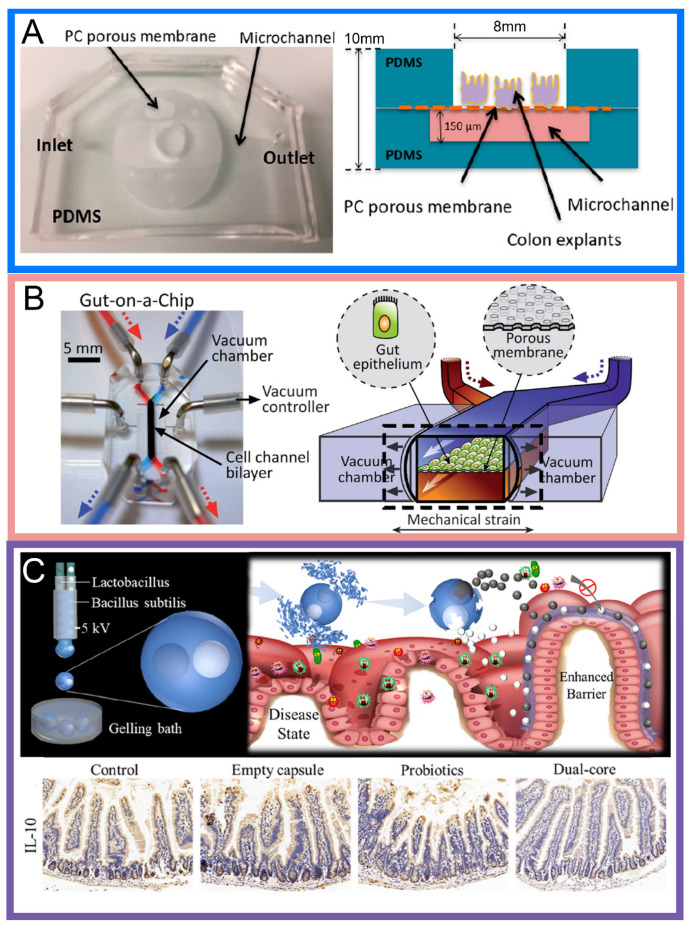
(**A**) Schematic and image of a microfluidic bioreactor [39]. Reproduced with permission from [39], 2020, MDPI AG. (**B**) Schematic and image of a gut-on-a-chip device with additional peristaltic motions using vacuum [81]. Adapted with permission from [81], 2012, Royal Society of Chemistry. (**C**) Schematic of microcapsule fabrication and release of probiotics within GI tract. Image comparison of probiotics influence with dual-core microcapsule fabrication showing lower intestinal inflammation for dual-core capsules [82]. Adapted with permission from [82], 2020, American Chemical Society.

***Microfluidic bioreactors***: Conventional bioreactors consist of multiple large beakers connected to a tube for the flowing of culture media or reagents of interest. They are designed and developed to provide a system that contains a controlled environment that is capable of mimicking a biologically active environment whereby cells or bacteria are able to thrive and interact [79]. Initially without any host organisms, these huge tanks are then loaded up with culture media and cells or bacteria. Single microbe cultures and/or microbiomes can thus be cultured within these beakers with some also consisting of integrated stirrers for mechanical stimulations [12]. Microfluidic bioreactors (MFBs) here miniaturize the entire setup into smaller individual chambers for such purposes (See Figure 3A). It also combines the advantage of conventional 2D and 3D methods with features from other bioreactor platforms, such as the induction of hydrodynamic forces. MFBs also offer improved controls and observations of microenvironmental conditions through integrated sensors and increased throughput, while finally reducing the volume of reagents needed [12,80], Especially for gastrointestinal microbes, there are additional requirements like the need for low oxygen levels (anaerobic settings), additional purification of samples (for stool sample processing), and a reduction in contamination and infections from the environment. A list of the advantages and disadvantages are mentioned in Table 2.

A mini-bioreactor assay (MBRA) was also developed by Auchtung et al. [83] in 2016 to study the dynamics of *Clostridium difficile* infection in the presence of fecal microbial communities. This setup was able to operate 48 small-volume reactors (up to 500 mL) in parallel within a single anaerobic chamber, effectively reducing the volume of reagents needed. Constant flow was also established with the use of peristaltic pumps that continuously pumped reagents through the reactors. This same setup was also used with a working volume of 15 mL per reactor, up to 24 reactors, for the evaluation of the antibiotic clindamycin and polyphenol extracts of blueberry and pomegranate PE on fecal microbiota [78]. The multiplexing ability there enabled the running of eight types of combinations in triplicates, reducing the time needed for experiments. Being an intermediate platform, such MBRAs have greatly improved the outlook for downsizing experimental setups. Therefore, they promote a greater need for microscale bioreactors.

Microfluidic bioreactors have been used in probiotics studies for observing bacteria growth preferences of *Lacticaseibacillus rhamnosus* GG [84], *Escherichia.coli* probiotic strain M-17 [85], and *Phaeobacter inhibens* DSM 17,395 strain [86]. A series of mini-bioreactors were fabricated within a microfluidic evolution on a chip (EVoc) for trapping of the bacteria *Lacticaseibacillus rhamnosus* GG within a channel containing an increasing level of H_2_O_2_. Observations recorded that a progressive exposure to H_2_O_2_ led to an evolution of the wild-type bacteria causing them to survive under previously intolerable environment [84]. A microfluidic chamber retrofitted from a counting chamber was developed for observations of bacteria motility for the M-17 strain, revealing two bacterial motility patterns, vertical and horizontal, and for adequate adhesion as well [85]. The directional motion of the bacteria was hypothesized to have huge implications on the surface colonization of the bacteria within the microfluidic chamber. A commercial microfluidic chip from Agnitio was also used to investigate the efficacy of taking personalized probiotics like the *Phaeobacter inhibens* DSM 17,395 strain on natural killer cell activity [86]. Unfortunately, the results published were insufficient to prove a causal relationship between the probiotic consumption and NK cytotoxicity. However, the study demonstrated that the IFN-γ/IL-10 value could be a potential biomarker for the evaluation of probiotics.

***Microcapsule fabrication devices***: Microfluidic capsules, as the name implies, are small pico/femtoliter storage compartments fabricated from the interaction between three or more types of fluids [87]. The fluids used are mainly comprised of an oil- or water-based continuous phase, a discrete phase containing target drugs or bacteria, and a shell phase that either cures chemically or through external activation. These capsules can be formed either through the use of a complex microfluidic device or through simple agitation within a bioreactor with both platforms having their own benefits [88]. However, the former method is still much preferred as it provides a more consistent capsule size and concentration albeit requiring a more complex equipment setup. A recent search on Scopus using the keywords “microcapsules” and “cancer immunotherapy” revealed that there was an increase from an average of 1.5 publications prior 2015 to 48 publications in 2023, indicating an increasing interest in this arena (See Supplementary Materials Figure S1).

Microcapsules play an important role in the protection of their contents from harm within the gastrointestinal tract. Being a means of travel across the GI tract, such capsules play an important role in cells co-culturing. This is especially important for cancer immunotherapy involving the prescription of probiotics and targeted drugs as these contents are mostly only effective at certain locations of the GI tract (see Figure 3C). If the contents are released before or after the specified location, the contents may either be destroyed by gastric juices or absorbed by other healthy cells that render the treatment ineffective. By encasing the contents with a protective barrier, it provides functions like the timed/gradual release of contents, or location-specific release of contents [8]. In vivo, this helps in the targeted delivery of drugs or microbiome. Chen et al. demonstrated the effects of an encased synthetic consortium of BsS-RS06551 and JJ3 using mouse trials which showed the increase in *Bifidobacterium longum* and the promotion of the gut vitamin B6 metabolism [89]. Ex vivo, this is also beneficial for the investigation of drug concentrations for different treatments and evaluation of gut microbiome interactions. Long et al. developed double-layered microcapsules using calcium alginate and gelatin for the timed release of drugs upon encountering simulated gastric fluid, and simulated intestinal fluid [90], Okra oil [91], surfactant-free oil [87], and fruit juice were also separately used as carrier fluids [92] for the production of such microcapsules, demonstrating the wide variety of encapsulation methods suitable for such applications.

***Gut-on-a-chip Devices***: A gut-on-a-chip (GOC) device is a microfluidic device designed to mimic intestinal morphology while providing an environment that allows users to replicate the physiology and interactions in the intestine [20,33]. GOC devices are used in the biomedical field for several purposes including drug testing, disease modeling, nutrient absorption studies, and microbiota interactions [49,93]. Cells are first grown within a microfluidic chip before the addition of subsequent reagents or bacteria cultures of interest. These cells are grown on a membrane layer, like the cell culture insert-type device, with the exception that the layer is fabricated together within the device. Being fully customizable and versatile, a GOC device may vary in design complexity while also allowing different adjustable parameters to be applied to it. Some examples of the adjustable parameters are cell types, culture condition, fluid flow rates, the presence of peristaltic motions, and host–microbe crosstalk [94].

GOC devices have previously been used for both 2D and 3D cell cultures, where 2D generally refers to single monolayer cultures. Three-dimensional cell cultures, on the other hand, include 3D structures or 3D scaffolds that mimic the villi and crypts of the intestinal epithelium, offering a more realistic model for experimentation. It is often understood that 2D models do not accurately represent the human intestine in vivo as it lacks crucial elements such as host–microbe interactions, fluid flow, peristaltic movement, and the complex cellular or tissue architecture. The further inability to form intestinal mucus and carry out cytochrome P450-based metabolism was also previously mentioned by Roodsant et al. [95]. When a comparison was made between Caco-2 cells formed on a 3D collagen scaffold versus a 2D collagen scaffold, it was seen that the permeability was higher for the 3D than the 2D one [96]. Another observation was that the Transepithelial Electrical Resistance (TEER) value of Caco-2 cells cultured on both 2D and 3D collagen scaffolds was observed to be lower than Caco-2 cells cultured on a semipermeable membrane of the conventional transwell model [96]. Therefore, experimental results suggested that the gut epithelium cultured on the 3D scaffold achieved the highest permeability, resembling the in vivo gut epithelium more closely [96]. Nevertheless, foundations and insights on gut microbiology can still be obtained by using such 2D cultures, which would also be applicable for 3D models.

The villilike structures that are characteristic in 3D cell culture GOC devices have been observed to affect the physiological intestinal functions such as drug absorption, mucus production, permeability, and the ability to resist invasion by pathogens or harmful bacteria invasion [29,97]. They are also crucial for maintaining the structural integrity of the intestinal epithelium [98]. Because of this, it is perceived that an ideal GOC device with a 3D cell culture structure should possess several critical characteristics which includes a 3D villilike structure that undergoes a peristalsis-like motion, shear stress, and mass transportation [49] (see Figure 3B). In addition, to ensure optimal conditions, 3D villi structures within GOC devices should encompass tight junctions, apical brush borders, mucus production, increased drug metabolizing activity, and enhanced glucose uptake [99]. Kim et al. developed a novel multichamber microfluidic device for the in situ culturing of gut epithelium cells atop a membrane middle layer that was used as a scaffold. On-demand peristaltic motion functions were also integrated with the use of vacuum chambers microfabricated alongside [81,100]. Comparisons with the traditional transwell insert methods also showed a heightened activity of drug metabolizing the cytochrome CYP3A4 enzyme that promotes villi formation, which was attributed to the physical microenvironment within the device itself [99]. Hybrids comprising GOC devices with transwell inserts have also been used, allowing a continuous flow while having easily removable culture layers [13] (see Figure 2D).

Shim et al. discovered that by comparing a 3D scaffold made of collagen with 2D cells on a monolayer, an improved metabolic activity was seen in the 3D cell culture [101]. However, under the same flow rate, there was a potential increase in shear stress at the top of the villi, and it could cause damage to the cells [101]. Through experimentation, it was observed that 3D villilike structures were formed within 3 days of culture in a microfluidic GOC device with or without addition of mechanical cyclic strain [102]. In another experiment, it was shown that when Caco-2 cells were cultured for >100 h, the cells spontaneously formed 3D villilike structures [99]. Similarly, Caco-2 cells were cultured in a GOC device, a planar epithelium was observed over the first 2 days of culture, and then it spontaneously transformed into 3D villilike structures over the next 1–2 days [99]. Similarly, Sung et al. used laser fabrication and sacrificial molding techniques to create micrometric hydrogel structures with high aspect ratios and curvature for the growth of Caco-2 villi structures [103]. It was further integrated into a microfluidic GOC model six years later to reveal that there was further differentiation and improved physiological relevance and metabolic activity under fluidic shear stress conditions [101]. This was also in accordance with another publication where the presence of shear stress within microchannels induced greater Caco-2 epithelial cell heights [81].

As mentioned earlier, the fabrication process for such microfluidic devices may be considered difficult to an untrained individual, being considered complex, costly, and time-consuming [77]. Thus, new ways to fabricate such devices have also been discovered with the most prominent being 3D printing.

**Table 2:** Advantages and disadvantages of conventional microfluidic devices [8,12,29,80,93,95,97,99].

Advantages	Disadvantages
Microfluidic Bioreactors High throughput Improved controls Reduces amount of reagents Observance of microenvironment Microcapsule Fabrication Devices Small volume Protected content Timed/gradual content release Location-specific content release Gut-on-a-chip Devices Improved permeability Enhances cell differentiation Allows physiological motions Improved enzyme and metabolic activity	Microfluidic Bioreactors Costly Requires trained expertise Complex setup Microcapsule Fabrication Devices Costly Highly reagent-dependent Difficult to achieve uniform coating Reaction between core and shell material Gut-on-a-chip Devices Requires additional equipment Requires trained expertise Introduce mechanical stress to cells Two-dimensional models lack crucial in vivo features Incomplete intestinal representation

### 2.3. Three-Dimensional Printing

Three-dimensional (3D) printing, also referred to as additive manufacturing, has revolutionized the manufacturing process by enabling the construction of intricate structures simply from a design file [42]. In the biomedical field, 3D printing holds significant importance for the advancement of tissue models and organ-on-a-chip devices, including the GOC devices. Being a simple, cost-effective prototyping method for devices with customized complex microfluidic features, 3D printing has provided an alternative solution for resolving persistent issues related to conventional microfluidic products [104,105]. A list of advantages and disadvantages are mentioned in Table 3. However, careful considerations are still needed with the choice of printers or material selection due to issues like surface roughness, material biocompatibility, and cytotoxicity, which can affect fluid flow and potentially lead to inadequate cell adhesion or cell death [104,106,107,108]. Readers are also advised to not confuse this with 3D scaffolding, which involves methods used for the fabrication of devices in the earlier sections like mold casting [109]. A list of commercial 3D printing technologies includes inkjet printing [104,110,111] (Systemic Bio [112]), polyjet printing [104,113] (Proto3000 [114], Stratasys [115]), stereolithography [42,104,116,117] (Miicraft [118], B9Creations [119], Kloe [120]), and fused deposition modeling [104,121,122] (Makerbot [123], Prusa Research [124], Ultimaker [125]).

**Figure 4:**
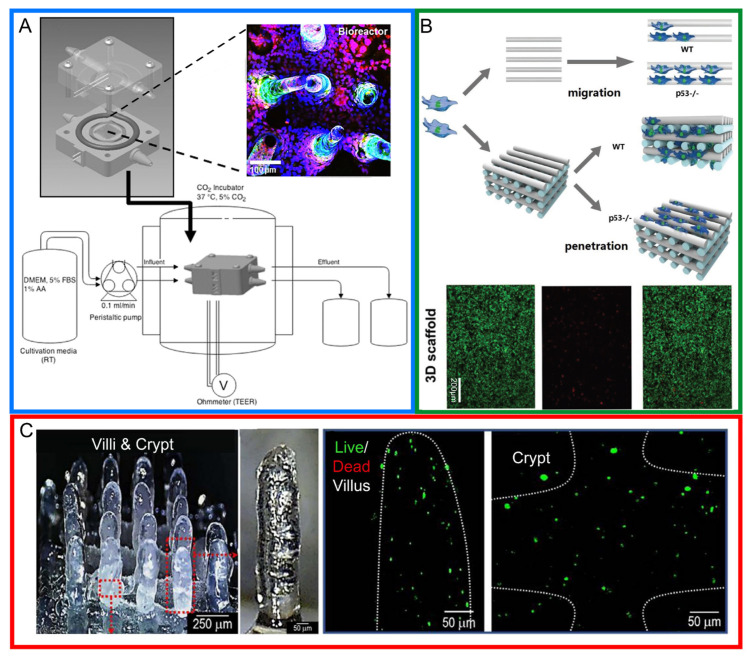
(**A**) Schematic diagram of a 3D-printed scaffold and vessel with villi for Caco-2 cells. Reproduced with permission from [79], 2017, Open Access. (**B**) Schematic diagram of a 3D-printed scaffold for human colon cancer cell migration evaluation. Reproduced with permission from [127], 2018, John Wiley and Sons. (**C**) Photograph and schematic diagram of 3D bioprinting showing villi and crypts with live/dead cell observations of red box locations [43]. Reproduced with permission from [43], 2024, Elsevier.

***Three-dimensional scaffolding***: Current 3D scaffolding methods involve using 3D printing technology to create a villouslike structure that resembles the human intestine. Once the structure is printed, cells are cultured on it and allowed to grow to further mimic the intestinal environment. Costello et al. used 3D-printing technology to produce an intestinal villouslike structure with polyvinyl acetate (PVA) and integrated it into a bioreactor to support cell culture [41] (see Figure 4A). The device allowed cells to be cultured for 32 days, and the intestinal cells showed similar in vivo characteristics in terms of nutrient absorption. Another similar platform developed by Taebnia et al. was also able to culture Caco-2 cells for a month while using SLA printing [126]. The novelty in this case was its printing of two types of poly(ethylene glycol) diacrylates (PEGDA) to produce cyto-compatible scaffolds. Human colon cancer cell lines HCT-116 and LoVo were also successfully cultured on a 3D-printed scaffold using electro-hydrodynamic jet printing for observations of tumor protein 53 (p53) on cancer cell motility and migration [127] (see Figure 4B). The electrospinning of polylactic acid (PLA) nanofiber scaffolds on a modified transwell was also demonstrated for the co-culturing of different probiotics (*L. plantarum*, *L. reuteri*, *B. infantis*, and *L. casei Shirota*) on Caco-2 cell monolayers showing a surprising greater adhesion of *B. infantis* as compared with the other probiotics, suggesting a differing physiological status of the scaffold with the traditional transwell models [128]. The 3D printing of reverse molds using polydimethylsiloxane (PDMS) was also utilized to form silk villi and crypts for co-cultures of Caco-2 and HT29-MTX cells [98].

***Three-dimensional bioprinting***: Another method of application for 3D printing involves the direct printing of bioinks such as collagen, extracellular matrix (ECM), and hydrogel [104] to print living cells, creating structures layer by layer. This is also often termed as 3D bioprinting [43,103,129]. It allows cells, biomaterials, and bioactive molecules to be positioned with precise spatial control, thereby mimicking the structures of a natural tissue [117,129]. A list of bioprinter technologies are categorized as inkjet-based printing (IBP), light-based printing (LBP), and extrusion-based printing (EBP) as effectively categorized by Zhang et al. [129]. Each printer type has its advantages and disadvantages and must be chosen based on specific requirements like speed, precision, and resolution. Kim et al. used 3D-bioprinting technology to print villilike structures using Caco-2 collagen ink crosslinked with tannic acid [43] (see Figure 4C). The use of high resolution stereolithographic 3D printing in that case enabled the generation of intricate microfluidic devices at a lower cost and a shorter fabrication time as compared with other fabrication techniques [43]. Biocompatible hydrogel-based scaffolds were also fabricated to mimic in vivo intestinal function and structure [126].

## 3. Future Advancement

With the implementation of rapid prototyping technologies and the continual importance of microfluidics in cell cultures, more efficient and effective design-to-device pipelines can thus be generated for more biomedical applications. One noteworthy point to mention would be the possibility of scaling up current capabilities for multiplexed drug testing. With the currently available microfluidic devices, it is possible to embark on the broad-spectrum testing of different drugs. However, there is still much to discover in identifying the effects of certain drugs on the different systems in the body. Present research has barely reached around three micro-physiological systems, showing that improvements still need to be made for a multi-system evaluation of different drugs [20]. As such, many revelations are still awaiting discovery in this aspect of drug testing. Another aspect that is close to realization is along the aspect of directional cell growth using 3D-printed materials. A recent summary of the fundamental knowledge in this field is provided by Zieliński et al. [130] with evaluations of type of printers, physical and biochemical parameters for controlling the growth direction. At present, directional electrospinning of nanofibers has been observed with improvements in cell growth; however, qualitative directional cell growth has yet to be reported [131,132]. Apart from this, we further discuss a few trending topics in the biomedicine arena related to cancer immunotherapy and how microfluidics would benefit such research.

### 3.1. Stool-Derived In Vitro Communities (SICs)

One approach to tackling issues in cancer immunotherapy is through the evaluation of how our daily food intake affects our body. It is often mentioned that “We are what we eat”, and this has also been scientifically proven through many outstanding discoveries in gut microbiome and gastrointestinal system research [133]. One means of investigating gut microbiome is through the use of stool-derived in vitro communities (SICs), otherwise known as feces-derived communities, or stool-derived communities [134]. It was reported that select gut microorganisms could act as biomarkers or targets for improving antitumor immunotherapy efficacies [135] and also as inhibitors or cancer promoters for gastrointestinal cancer [136]. SICs are thus able to assess the genetic diversity of an individual’s microbiome while also providing personalized insights for drug–microbiome evaluations [137].

Presently, the gold standard protocols for SIC culturing is still using small beakers as bioreactors like the Robogut [138,139] or screening plates [133,134,137] that are cumbersome and with multiple steps in between. Continuous flow culture models have also been demonstrated; however, they are still lacking in throughput [133]. An ideal situation would consist of the fully automated handling of all samples and reagents, which is where the microfluidic systems come into play. Unfortunately, much more engineering and innovations are still needed for this to be possible as the gut microbiome cultured here are more complex than normal co-cultures [137]. Detection mechanisms for each individual bacteria type also need to be considered carefully while still ensuring stable and optimal environments for growth. Sorting of each bacteria type may also be more appropriate before final reconstitutions, which would also require more complex microfluidic structures.

### 3.2. Gut–Immune–Tumor Research

Gut microbiota have been used for investigations relating to antibiotics studies [138] and various cancer studies like breast cancer [140,141], pancreatic cancer [141], and colorectal cancer [141,142]. It is no surprise to observe such huge implications of these microbial communities within our body, revisiting the same quote mentioned earlier. The gut microbiome regulates the immune system through various means, which is briefly summarized by Zhang et al. [135]. Although this application is well researched, with numerous publications mentioned here, it is observed that the technology used here is relatively dated and does not have the benefits brought about by microfluidic devices.

Some challenges in gut–immune–tumor (GIT) axis research include the need for culturing multiple types of cells that would accurately reflect the human gut physiology. Firstly an intestinal mucosal barrier consisting of a layer of intestinal epithelial cells is needed, separating the immune cells from the microbiome environment within the gut [143]. Subsequently, another system comprising tumor or stromal cells also need to be cultivated where vascular endothelial cells are then separately cultured for both systems. The culturing of these multiple types of cells is tedious with each system requiring different sets of reagents and environmental conditions (like media acidity and oxygen levels) for successful growth. These general considerations further increase the difficulty of developing GIT microfluidic devices. A list of cancer-on-a-chip devices was mentioned in a recent paper by Safhi et al. [144] using 3D printing. Although the context was smart orthopedic implants, it may be possible to integrate such smart materials in microfluidic devices that can be subsequently used as wearables for long-term clinical detection of cancer cells in situ. Four-dimensional printing was also mentioned, using shape memory polymers and smart hydrogels, preventing accidental damage to the chip with its self-repairability function, and allowing robust packaging of highly sensitive devices. Another 3D-printed scaffold for cancer cell culture was developed by Rosendahl et al. where a novel material, TEMPO-CNF, was introduced [145]. Their work demonstrated that the cancer cells were able to fill out the pores and cavities within the scaffold, mimicking in vivo situations. In 2021, Trapecar et al. [14] developed a micro-physiological system comprising the gut, liver, and brain for neurodegenerative diseases. This would seem to be the closest step taken with a mesofluidic device for three different biological axes. However, further improvements are still required to fully develop it into a microfluidic, self-contained environment.

### 3.3. Modular Microfluidic Devices

An interesting concept in the engineering aspect of microfluidics discusses the integration of different modular components on demand for additional functionality [146,147]. Microfluidics being versatile in nature holds a strong challenge in that it requires detailed planning before fabrication with different functional parts included at the designing stage. Further additions to the chip require redesigning and refabrication of device molds altogether. Modular microfluidics thus eliminates this need, as each module is considered as an attachment that can be integrated into the device on demand. A variety of platforms have been developed like the Lego^®^ design [148,149], microfluidic building blocks (MFBB) [150], and Tetris-like (TILE) microfluidic platform [151]. A recent study by Lai et al. [146] in 2022 revealed that there was an increasing interest in modular microfluidics. However, its uptake as a cell culture platform is relatively stunted. Some main reasons could be due to the risk of leakages or difficulty in the fabrication of such platforms. Leakages present a major issue in biological experiments due to the risk of contamination or exposure to the environment and thus human health. As such, strict requirements are enforced for such applications. The fabrication of these modular platforms involve unconventional means of fabrication within the microfluidic field, with one example being the use of superthick photoresist films [148] or complex infrastructure for the casting of magnets within the chip [151]. Upon resolving such issues, it is envisioned that such modular microfluidic devices would be great cell culturing platforms.

The use of such platforms would firstly provide much needed flexibility for users. Cells would be cultured on one modularized platform and removed for easy attachment onto another platform for the direct introduction of drugs and reagents of interest. Current methods of microfluidic cell culturing involve much discernment with the balancing of fluidic pressures to ensure that cells are seeded at specified locations within the microfluidic device. This is due to the need for multiple separate channels for the subsequent introduction of reagents, like the device by Virumbrales-Muñoz et al. [152]. By modularizing the device, only a simplified design is needed for the cell culture procedure, where subsequently, the culture can be detached and integrated into another section, analogous to how the space station was built. Another benefit is the multiplexing of cell cultures. Current methods of multiplexing cell cultures in microfluidics is through the use of a myriad of channels connected to individual bioreactors, like the device by Trapecar et al. [14]. The amount of multiplexing is restricted to the number of chambers designed into it. With modularized components, extensions to the platform can be carried out with ease, enabling many more experiments to be conducted simultaneously. With these being some of the advantages to the use of modularized microfluidic devices, it is believed that cell cultures would thus be carried out with higher efficiency and throughput.

### 3.4. Point-of-Care Applications

Another prominent trend in research points towards the direct usage of analytical tools right beside a patient/user, also termed as a point-of-care (POC) device. POC devices help to provide fast, reliable access to laboratory data, improving care and patient outcomes with timely clinical decision making [153]. These devices have been readily used in different contexts with the discovery of biomarkers for different cancers [154] and diseases [155] via a variety of clinical samples like blood, saliva, urine, and stool. However, in the context of cell culturing technologies, a large barrier remains pertaining to the actual cultivation of cells within the device. Time is required for the formation of a monolayer of cells within the device, therefore inhibiting its use as a POC device. Nevertheless, multiple biosensors have been developed for the detection of GI cancer and constituents of its microbiome.

In 2015, a review by Huddy et al. [156] established that POC techniques had already been used for patient screening for gastrointestinal cancer through the detection of constituents like fecal occult blood, calprotectin, volatile organic compounds (hexanoic acid, phenol, methyl phenol, and ethyl phenol), pyruvate kinase isoenzyme type M2 (M2-PK), circulating tumor cells, and DNA biomarkers like SEPT9 and tumor protein (p53). However, it was also specifically mentioned that there was a strong lack of evidence supporting the reliability of these works coupled with a strong need for the pre-treatment of certain samples like stool, for example. Another more recent series of biosensors and bioelectronics was compiled by Ngashangva et al. [157] in 2023 demonstrating the detection of gastrointestinal microbes and gut metabolites. However, it was also mentioned that such biosensors produced a high variability in the results despite similar diseases, revealing the complexity of gut microbiota relationships and biomarkers. Ultra-sensitivity and specificity may therefore need to be the primary criteria for biosensors before enabling actual use as POC devices. With this sorted out, subsequent installations of sample pre-treatment components would thus provide a highly effective device for clinical use.

A closest relation to POC devices was given in this recent list of commercially available lateral flow assay paper-based microfluidic kits presented by Bhardwaj et al. [154] for cancer detection in general. For GI-related devices, the main samples required are blood and stool samples, whereby pre-treatment of samples must still be performed manually. The integration of microfluidic channels to resolve such issues remains a challenge. Although many different methods of detection have been established using optical and electrochemical means, discussions mentioned by Behera et al. [158] on integrated microfluidic devices for POC detection of bio-analytes and diseases also reveal that there still remains a strong lack of application on the GI tract. It is therefore conclusive to state that much work still awaits to be carried out in developing microfluidic POC devices for gastrointestinal applications. Upon successful development, subsequent integration with cell culture technologies would therefore provide more insights for clinical diagnosis and immediate treatment solutions for cancers and diseases.

## 4. Conclusions

Gastrointestinal cell culture technologies have made tremendous improvements in the past years when used in conjuncture with microfluidics. Transitioning from huge vats to small microfluidic platforms, more specific and physiologically relevant discoveries can be made. These platforms were categorized into three types, namely cell culture insert devices, conventional microfluidic devices, and finally, 3D-printed microfluidic devices. With each of them different in nature, the ease of usage also gradually decreased, where the latter few required more technical expertise. Nevertheless, the uptake of the latter few methods were still high due to the ability to include additional sensors to detect the microenvironment, coupled with modifications to introduce dynamism into the system and increased design sophistication. Gut-on-a chip devices and microcapsule fabrication devices have also been used for co-culturing probiotics and in drug delivery applications, which are both trending topics at present. The further integration of microfluidics in topics like stool-derived in vitro communities and gut–immune–tumor axes would prove to be of great benefit, as there is still a lack of representation. The use of modular microfluidic components would also be greatly appreciated if issues like leakages and fabrication complexities could be resolved. Finally, the use of GI cell culture technologies as point-of-care devices still remains challenging to date although multiple breakthroughs have been established since a decade ago.

In conclusion, microfluidic cell culturing devices for gastrointestinal tract investigations have greatly benefitted the scientific community with multiple options to cater to the different needs of different applications. Soon, it is hypothesized that these technologies can also be applied to more diverse applications through the breaking down of traditional barriers. Simpler models with more comprehensive solutions can also be developed for these trending research areas mentioned here, greatly benefiting the scientific community.

## Figures and Tables

**Table 3:** Advantages and disadvantages of 3D printing [42,43,102,103,104,105,106].

Advantages	Disadvantages
3D Scaffolding/3D Bioprinting Simple operation Cost effective Shorter fabrication time Able to produce complex design	3D Scaffolding/3D Bioprinting Surface roughness Material biocompatibility Cytotoxicity

## Supplementary Materials

The following supporting information can be downloaded at: https://www.mdpi.com/article/10.3390/bios14090449/s1, Table S1: Chip Preparation.
