# Supplementary material for: Microfluidic Gastrointestinal Cell Culture Technologies—Improvements in the Past Decade
Source: Biosensors (Basel). 2024 Sep 19;14(9):449. doi: 10.3390/bios14090449 (PMC11429516; doi:10.3390/bios14090449)
Supplement: Supplementary file 1 [file biosensors-14-00449-s001.zip › biosensors-3159432-supplementary.pdf]

Table S1 - Chip Preparation

The microfluidic chip described here is prepared in Nanyang Technological University (NTU) main campus laboratory and the detailed steps for the chip preparation process is documented in this section. The full protocol mentioned here is for the preparation of a PDMS microfluidic gut-on-a-chip device.

For standard microfluidic devices using PDMS, the crosslinking ratio in Step 1 can be modified to 10:1. Steps 9-19 may be ignored while the term “Bottom PDMS” from Step 20 onwards may be considered as the “PDMS piece” with the indented microfluidic channels. Step 23 will therefore be “Assemble the PDMS piece and a glass substrate together.”.

The equipment used here is an air plasma generator (Harrick Plasma Inc, PDC-32G), and an incubator (HCS Scientific & Chemical Pte Lts, INCUCELL).

Step 1 to 7 describes the PDMS Preparation Process.

Step 1	**PDMS Elastomer Mixing**
a) Cross-Linking SYLGARD 184 (Silicone Elastomer Base and Silicone Elastomer) with the ratio of 15 : 1.<br><br>b) Whisk the mixture well to ensure that the elastomer is evenly mixed.<br><br>c) Pour the whisked mixture into a prefabricated mold and ensure that the mold is fully filled.	

Step 2	**Vacuum**
	To remove the air bubbles, place it in a vacuum chamber.
Duration : 30 to 60 minutes.	

Step 3	**Incubation**
	Place in an incubator to ensure that the PDMS cures.
Duration : 90 minutes<br>Temperature : 70 Degree celsius.	

Step 4	**PDMS (both Top and Bottom) Removal**

Carefully cut out the PDMS from the mold.

Step 5	**Perforate Holes for Top PDMS**
	Holes have been punched on the PDMS to allow fluidic flow through the chip.

Step 6	**Clean PDMS (both Top and Bottom)**
	To prevent contamination, the PDMS is washed with IPA and cleaned dry with an airgun.

Step 7	**Incubate PDMS (both Top and Bottom)**
	Place in an incubator to ensure that the PDMS is dried entirely.
Duration : 30 minutes<br>Temperature : 70 Degree celsius.	

Step 8 to 14 describes the membrane preparation process.

Step 8	**Membrane Preparation**
	Carefully cut the membrane to a suitable size to accommodate the chip size.

Step 9	**Plasma Activate Membrane**
	The membrane is plasma activated with the air plasma generated using a Harrick plasma cleaner.
Duration : 2 minutes<br>RF Level : High<br>Vacuum setting: ON	

Step 10	**Chemical Preparation for Membrane**
Prepare the respective chemicals and ensure that the final composition is well mixed.	

Chemicals	Quantity (microliters)
Aminopropyl Triethoxysilane	40
Titanium (IV) butoxide	10
IPA	2000

Step 11	**Soak Membrane**
	Soak the membrane in the solution prepared earlier on a petri dish.
Duration : 20 minutes<br>Temperature : Room Temperature	

Step 12	**Wash Membrane**
	After 20 minutes, wash the membrane by diluting the mixture with IPA.

Step 13	**Incubate Membrane**
	Place in an incubator to ensure that the membrane is dried entirely.
Duration : 30 minutes<br>Temperature : 70 Degree celsius.	

Step 14	**Soak Membrane**
	To allow the membrane to be hydrophilic, 70% ethanol is applied.
Duration : 30 minutes<br>Temperature : Room Temperature	

Step 15 to 23 describe the assembly process.

Step 15	**Plasma Activate Top PDMS**
	The membrane is plasma activated with the air plasma generated using a Harrick plasma cleaner.

Duration : 2 minutes<br>RF Level : High<br>Vacuum Setting: ON

Step 16	**Attached Membrane**
	Membrane is placed onto the PDMS top surface.

Step 17	**Perforate Holes for Membrane**
	Holes have to be punched on the membrane to allow fluidic flow for the bottom microfluidic channel.

Step 18	**Incubate the Top PDMS with Membrane**
	Place in an incubator to ensure that the PDMS is dried entirely.
Duration : 60 minutes<br>Temperature : 70 Degree celsius.	

Step 19	**Clean Bottom PDMS**
	To prevent contamination, the PDMS surface is washed with IPA and cleaned dry with an airgun.

Step 20	**Incubate Bottom PDMS**
	Place in an incubator to ensure that the PDMS is dried entirely.
Duration : 30 minutes (estimate)<br>Temperature : 70 Degree celsius.	

Step 21	**Plasma Activate Bottom PDMS**
	The membrane is plasma activated with the air plasma generated using a Harrick plasma cleaner.
Duration : 2 minutes<br>RF Level : High<br>Vacuum setting: ON	

Step 22	**Final Assembly**
	Assemble the PDMS Top with Membrane and the PDMS Bottom together.

Step 23	**Incubate Device**
Place in an incubator to allow complete bonding of PDMS parts.	
Duration : 30 minutes (estimate)<br>Temperature : 70 Degree celsius.	
